# Impact of Climate Change on the Presence of Ochratoxin A in Red and White Greek Commercial Wines

**DOI:** 10.3390/foods14234157

**Published:** 2025-12-03

**Authors:** Dimitrios Evangelos Miliordos, Lamprini Roussi, Stamatina Kallithraka, Efstathios Z. Panagou, Pantelis I. Natskoulis

**Affiliations:** 1 Institute of Technology of Agricultural Products, Hellenic Agricultural Organisation (ELGO)—DIMITRA, 1 Sofokli Venizelou, 14123 Likovrisi, Greece; dim.miliordos@gmail.com; 2 Laboratory of Oenology and Alcoholic Drinks, School of Food and Nutritional Sciences, Department of Food Science & Human Nutrition, Agricultural University of Athens, 75 Iera Odos, 11855 Athens, Greece; lamrous98@gmail.com (L.R.); stamatina@aua.gr (S.K.); 3 Laboratory of Microbiology and Biotechnology of Foods, School of Food and Nutritional Sciences, Department of Food Science and Human Nutrition, Agricultural University of Athens, 75 Iera Odos, 11855 Athens, Greece; stathispanagou@aua.gr

**Keywords:** Ochratoxin A, Greek wine, QuEChERS, climate change, mycotoxins, HPLC

## Abstract

Wine samples (72) of different types (white, rose and red), dry, originating from different regions of Greece (Northern Greece, Central Greece, Peloponnese, Aegean Islands, and Crete), were analyzed for Ochratoxin A (OTA) presence. Wine samples, originating from Greek (Assyrtiko and Xinomavro) and international (Syrah and Sauvignon blanc) noble grapevine varieties vintaged from 2020 to 2023, were analyzed using a modified QuEChERS extraction protocol followed by HPLC with a fluorescence detector to detect and quantify OTA. Moreover, conventional oenological parameters were measured according to OIV official methods, and climatic conditions of the regions of concern were retrieved. Interestingly, in general, OTA contaminated wines showed low concentrations (<2.0 μg/L). The highest concentrations of OTA were detected in Sauvignon blanc (7.5 μg/L) regarding the white wines and Xinomavro (2.07 μg/L) regarding the red ones. In addition, the highest OTA concentrations were recorded in wines produced in areas either with high mean annual temperatures, like Viotia (24.16 °C) for white and Larissa (23.9 °C) for red wines, or with high rainfall between May and September (Larissa 69.76 mm) for white wines. Consequently, it was observed that concentrations of OTA in wine are relatively higher in the warmer regions of Greece compared to the cooler areas. The effect of climate change on vines and mycotoxin presence in wine needs urgent consideration by well-constructed modelling studies and efficient interpretation of existing data. The evaluation of OTA presence in grape products originating from various cultivars and regions is imperative not only for providing crucial data to predict its fate under climate change, but also to ascertain the potential risk of human exposure to this chemical compound.

## 1. Introduction

Europe represents the preeminent nucleus of viticulture globally, with Greece being among the top 20 nations possessing an area under vine exceeding 32 kha, specifically encompassing 106 kha of vineyards as of the year 2018 [1]. This designated area to produce wine, table, or dried grapes encompasses regions characterized by markedly diverse climatic conditions and agronomic methodologies, thereby yielding grape products with varying physicochemical attributes. The cultivation of vines flourishes in arid-thermal zones exhibiting substantial sunlight and minimal relative humidity; these environmental factors are instrumental in the production of wine with optimal organoleptic properties [2].

Crucial factors that are affected by these variable climatic and agronomic conditions include the possibility of contamination by several pathogenic fungi during berry growth, harvest, and post-harvest periods [3]. Filamentous fungi belonging to the genera *Aspergillus* and *Penicillium* can synthesize ochratoxin A (OTA) as secondary toxic metabolite. OTA is prevalent in various foodstuffs including cereals, legumes, nuts, spices, desiccated fruits, coffee, dairy products, beer, grapes, and products derived from grapes [4]. Following cereals, wine in some European countries is the primary source of daily OTA consumption [5].

OTA was initially identified in wines across various countries [6] and subsequently detected in grapes [7]. Climate change is recognized as a critical factor influencing the prevalence of OTA in grapes, along with the geographic location and health conditions of plants [8]. It is particularly noteworthy that winemaking techniques, such as maceration, can increase OTA levels in wines, resulting in its predominance in red wines, followed by rosé and white wines [9,10].

The concentration of OTA in wine is influenced by climatic conditions (notably, temperature and relative humidity in the pre-harvest month), viticultural practices (which encompass the application of fungicides), the proportion of damaged berries prior to maceration, and the specific type of maceration employed [11,12]. Research has indicated that the levels of OTA in wine are contingent upon the geographical latitude of the production area; wines sourced from grapes cultivated at lower latitudes exhibit a higher frequency and increased concentration of OTA contamination [11]. OTA has been implicated in various toxicological effects including nephrotoxicity, neurotoxicity, hepatotoxicity, teratogenicity, immunotoxicity, and carcinogenicity [13]. The International Agency for Research on Cancer (IARC) has classified OTA as a potential human carcinogen (Group 2B) [14]. Furthermore, the European Food Safety Authority (EFSA) has established a Tolerable Weekly Intake (TWI) of OTA at 120 ng OTA/kg of body weight [15]. This guidance value derives from a comprehensive risk assessment process conducted by EFSA, which takes into account the steps of hazard identification and characterization (including dose–response assessment), exposure assessment, and risk characterization, which will finally determine the potential adverse health effects associated with mycotoxin intake. Various regulatory agencies have used these principles to conduct risk assessments on main mycotoxins such as Aflatoxins (AFs), Deoxynivalenol (DON), Fumonisins (FBs), Ochratoxin A (OTA), and Zearaleonone (ZON), to set food safety guidelines [16]. The essential elements for the exposure assessment step are continuously updated prevalence and consumption data [17,18]. The overall process will in parallel supply the authorities with the critical information to set or update legislative maximum permissible levels (MPLs) of a mycotoxin in several food commodities. For instance, within the European Union (EU), the permissible maximum concentration of OTA in wine is 2.0 μg/L [19]. However, these safety limits are established for individual mycotoxins, and no consideration is given on the co-occurrence of mycotoxins, which could be of higher concern and may lead to substantial under-estimation of the actual health risk to consumers. Therefore, more comprehensive risk assessment studies focusing on combined exposure to multiple mycotoxins are in need [20].

Methods for extracting ochratoxin A (OTA) from biological matrices and subsequent sample preparation have been improved. The extraction and preparation phases have major limitations in mycotoxin analysis [21]. Notable advancements include solid-phase extraction (SPE) and the use of costly immunoaffinity columns (IAC) for selective OTA cleanup and enrichment prior to analysis [22,23]. A new trend in extraction and preparation is the adoption of “quick, easy, cheap, effective, rugged, and safe” (QuEChERS) methodologies and other optimized approaches [23]. Numerous studies have reported the development of simplified techniques, including “dilute and shoot” methods and those requiring minimal preparation [24,25,26,27].

Greece, like numerous other Mediterranean countries, has a rich winemaking tradition [28]. The Greek wine market is characterized by a plethora of monovarietal wines, attributed to a multitude of small-scale producers who produce limited quantities of wine, frequently utilizing not only esteemed international grape varieties, but also indigenous [29] and rare cultivars [30]. Nevertheless, to the best of our knowledge, there is a limited number of research reports concerning OTA concentrations in commercially available Greek wines [31,32,33,34,35,36,37], and crucially, no recent study has systematically investigated the temporal relationship between climate trends and OTA prevalence in Greek vineyards.

In light of the established toxicological ramifications of OTA, the dearth of comprehensive data regarding its concentrations in Greek wines, and their potential exploitation for risk analyses, this study aims to elucidate the prevalence of OTA in commercial Greek white and red wines and to analyze its potential relationship with recent climate trends.

Furthermore, it has been posited that the significant increase in OTA levels in wine over a decadal timeframe may be substantially influenced by climate change, thereby necessitating the ongoing monitoring of OTA concentrations in wine [38]. Thus, the present study will provide critical insights into the current state of natural contaminants in Greek wines and elucidate the specific attributes of these wines that may predispose them to the presence of the investigated mycotoxin.

## 2. Materials and Methods

### 2.1. Wine Sampling

To investigate the levels of mycotoxin contamination of OTA, 72 labels (bottles) of commercial wines were collected from wineries across Greece from 15 wine producing areas (Supplementary Figure S1). Among the 72 samples, 37 white wines (25 Sauvignon blanc and 12 Assyrtiko) and 35 red wines (23 Xinomavro and 12 Syrah) were included. All the selected wines were produced between 2020 and 2023. The samples were stored at room temperature in the dark until further use.

### 2.2. Physicochemical Analyses of the Wine

The principal compositional parameters of the vinous products, including residual sugars (RS), pH, titratable acidity (TA), volatile acidity (VA), and ethanol content (% Vol), were determined in accordance with the Compendium of International Methods for the Analysis of Wine and Must [39].

### 2.3. Sample Preparation and Mycotoxin Extraction of Wine Samples

The extraction of targeted mycotoxin from wine samples was performed according to the QuEChERS (Quick, Easy, Cheap, Efficient, Rugged, and Safe) method [37]. The methodology was delineated with precision: a 3 g sample of the wine was meticulously transferred into a 50-milliliter polypropylene screw-cap Falcon tube, followed by the addition of 7 mL of ultra-pure water. The mixture was briefly agitated, 10 mL of acetonitrile were added, and the resultant solution was vortexed for 1 min. Thereafter, 4 g of anhydrous magnesium sulfate (coarse), 1 g of trisodium citrate dihydrate, and 0.5 g of disodium hydrogen citrate sesquihydrate were added, and the sample was immediately vortexed for another minute. The sample was centrifuged at 5000 rpm for 5 min, and subsequently, 3 mL of the clear supernatant was extracted and placed into a clean 15 mL screw-cap Falcon tube containing 450 mg of anhydrous magnesium sulfate (fine). The sample was vortexed for 1 min, then centrifuged at 4000 rpm for 5 min, and 2 mL of the supernatant was transferred into a glass test tube. The sample was concentrated to dryness under a gentle stream of nitrogen and subsequently reconstituted in 400 μL of methanol (containing 0.1% formic acid) and 600 μL of water (containing 0.1% formic acid).

### 2.4. Chromatographic Separation

Ochratoxin A analysis was performed using reverse-phase High Performance Liquid Chromatography with fluorescence detection (HPLC-FLD). This consisted of a JASCO AS-4050 auto-sampler, a JASCO LC-Net II/ADC system controller, a JASCO PU-4180 pump, and a JASCO FP-2020 Plus fluorescence detector (JASCO Inc., Easton, CA, USA). The samples were separated using a C18 analytical column (250 × 4.6 nm, 4 mm, Resteck Co., Pinnacle II, Bellefonte, PA, USA) under isocratic conditions at a mobile phase flow rate of 1 mL/min (water/acetonitrile/acetic acid: 99/99/2). All chemicals used for HPLC analysis were of HPLC grade (methanol and acetic acid: Sigma-Aldrich Co., Darmstadt, Germany; water and acetonitrile: Carlo Erba Reactifs SDS, Val de Ruill, France). An excitation wavelength of 350 nm and emission wavelength of 450 nm were used for detection. Standard solutions were prepared from stock ochratoxin A solution (10.06 mg/mL in acetonitrile; Biopure, Romer Labs Diagnostics GmbH, Tulln, Austria), and a recovery study was conducted by spiking known concentration solutions (0.25, 0.5, 1, 2, 5, 10 μg/L) to wine and following the same extraction procedure as for the samples. The running time for the samples was 20 min, with OTA detected at approximately 8.7 min. The limit of quantification (LOQ) was 0.1 μg/L, while the limit of detection (LOD) was 0.03 μg/L (Table 1).

### 2.5. Method Validation

The method for determining OTA in wine samples was validated in terms of trueness, precision, linearity, and limit of quantitation. The Limit of Detection (LOD) and Limit of Quantification (LOQ) were determined based on the signal-to-noise ratio (S/N). The LOD was defined as the concentration yielding a S/N of 3:1, and the LOQ was defined as the concentration yielding a S/N of 10:1. Based on this approach, the LOD and LOQ for OTA in wine were established as 0.03 μg/L and 0.1 μg/L, respectively. Recovery was assessed by spiking OTA-free wine samples with standard solutions to obtain final concentrations of 0.25, 0.5, 1, 2, 5, and 10 μg/L. Each recovery experiment was performed in triplicate.

### 2.6. Meteorological Data

Meteorological data were systematically acquired throughout the experimental phase from the Automatic Network of the National Observatory of Athens [40]. Climatological data were exclusively compiled from the geographical regions where the wines tested positive for ochratoxin A (OTA). Meteorological parameters (Mean Temperature, Maximum Temperature, Minimum Temperature, Rainfall) were gathered from meteorological stations from May to September for the three years of vintages, given that the grape varieties transitioned to véraison leading up to harvest, a period during which they exhibited increased vulnerability to fungal infection by *Aspergillus* spp.

### 2.7. Statistical Analysis

All values are presented as the mean ± standard deviation. Statistical analysis was conducted using GraphPad Prism (version 8.0.1, GraphPad Software Inc., Boston, MA, USA). The significance of the results was determined using an unpaired *t*-test or one-way ANOVA with Tukey’s test for mean comparison. Additionally, multivariate statistical data analysis (MVA) of the samples was performed with SIMCA P+ version 15 (Umetrics AB, Umeå, Sweden) after mean centering all the variables and scaling to unit variance. GraphPad Prism (version 8.0.1, GraphPad Software Inc., Boston, MA, USA) was used for the correlations with the Pearson correlation coefficient (r) were used to study the linear relations between meteorological parameters (mean temperature, max temperature, minimum Temperature and rainfall) and the OTA concentration in wines. The statistical analysis results were considered significant when *p* < 0.05.

## 3. Results

### 3.1. Climatic Characteristics of the Wine Producing Area

In the present study, the climatic conditions of the viticultural regions were examined from May to September, given the established understanding that the prevalence of contamination by ochratoxigenic Aspergillus species is significantly diminished in immature green grapes, as these grapes provide an inhospitable environment for spore germination [41]. During the period from véraison to harvest, the berries exhibit heightened vulnerability to infection by OTA-producing fungi, attributable to their softened skin and increased sugar concentration, which are conducive to the colonization and proliferation of *Aspergillus* spp. [42]. Therefore, a postponement in the harvest of mature berries is associated with an elevated risk of OTA contamination [43]

The climatic characteristics pertaining to the various vineyard regions have been detailed in Supplementary Table S1, which specifically addresses the red grapevine varieties, whereas Supplementary Table S2 is focused on the white grapevine varieties. In a general overview, it was observed that the average temperatures recorded at mainland stations were significantly lower, particularly in the regions of Drama, Florina, where the mean temperature was approximately 19.76 °C, in contrast to the notably higher average temperatures found at Attiki (24.57 °C) and Viotia (25.04 °C), located in Central Greece, as well as the in Greek islands, the mean temperature reached a remarkable 24 °C on Lipsi Island and Crete and 23.76 °C on Tinos Island. Conversely, it is essential to highlight that the extremes of maximum temperatures, specifically the highest and lowest values, were noted at the mainland locations, with Larissa and Viotia exhibiting maximum temperatures of 38.54 °C and 38.5 °C, respectively, while the minimum temperatures were recorded in Florina (7.54 °C) and Achaia (7.78 °C). The recorded rainfall varied significantly, ranging from a minimum of 0.44 mm observed in Santorini to a substantial 118.76 mm noted in Drama. Rainfall exhibited a high degree of variability across all studied locations during the crucial period spanning from véraison to harvest for the examined grapevine varieties, as elucidated in Supplementary Tables S1 and S2. Notably, with regard to the red grape varieties, the highest levels of rainfall were documented in Florina, where a total of 52.6 mm was recorded during the 2020 vintage for the Xinomavro variety, while for the Syrah variety, the highest rainfall was observed in Viotia, amounting to 49.44 mm during the 2023 vintage (Supplementary Table S1). In relation to the white wines, the highest rainfall corresponding to the Sauvignon blanc variety was recorded in Achaia (72.4 mm) during the 2023 vintage, and for the Assyrtiko variety, in Drama (118.76 mm) during the 2020 vintage (Supplementary Table S2).

In terms of the origin of the sampled grapevine varieties, a definable gradient can be observed, characterized by increasing concentrations of OTA from the mainland regions (Florina, Larissa, and Viotia) towards the southern areas (Laconia and Irakleio).

### 3.2. Assessment of Mycotoxin Contamination Levels in Red and White Wines

A wide range of Greek monovarietal red and white wines were analyzed for OTA contamination. The samples represented geographically diverse sources, as they included wines from both continental (Eastern Continental Greece) and island regions of Greece (Crete, Evia, and Santorini), characterized by distinct climatic conditions. Eastern Continental Greece has a continental climate with hot summers, cold winters, and sufficient rainfall to make it a major agricultural area. The coastline has a Mediterranean climate, with hot and humid summers and mild winters. Furthermore, the investigated Greek wines can be traced by their protected designation of origin (PDO) to the wine-making sub-regions Santorini and Naousa. OTA concentrations in the analyzed wines ranged from 0.288 μg/L to 2.078 μg/L for Xinomavro and from 0.171 μg/L to 1.857 μg/L for Syrah wines, while the average and median values for Xinomavro (0.918 μg/L and 0.652 μg/L) were higher compared to the Syrah wines (0.804 μg/L and 0.580 μg/L) (Table 2), respectively, with only 4 wines having an OTA concentration exceeding 2 μg/L in Xinomavro and 2 in Syrah wines, respectively (Table 2). Concerning Assyrtiko and Sauvignon blanc, the number of wines detected OTA positive above the regulatory limit of 2 μg/L were 2 and 5, respectively (Table 2).

Of the 72 studied Greek wine samples (white and red wines), 40% were positive for OTA contamination. Even though the percentage of positive samples indicated a high incidence of contamination (55%), the majority of positive samples assayed (90.3%) presented low levels of OTA. Furthermore, only 8 samples exceeded the EU permissible maximum level of 2.0 μg/L (Supplementary Tables S1 and S2). Among the red wines, Xinomavro presented more OTA positive wines (7 wine samples) with one sample exhibiting more than 2 μg/L OTA compared to the international variety of Syrah, where 5 positive OTA wines were identified, with no wine samples exceeding the EU permissible maximum level of 2.0 μg/L.

In contrast, white wine showed a higher OTA incidence. For instance, the international variety Sauvignon blanc recorded 10 positive wine samples (83%) out of 12 tested wines, whereas for the Greek variety Assyrtiko, 18 samples were identified as OTA positive out of 25 (72%) (Table 2). The Sauvignon blanc variety presented a higher number of OTA positive samples exceeding the EU permissible maximum level of 2.0 μg/L compared to the Assyrtiko wines.

### 3.3. Conventional Wine Analysis and OTA Levels

**Figure 1:**
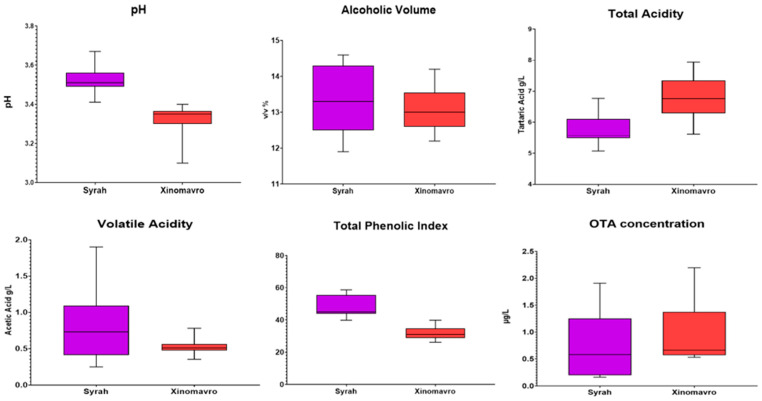
Boxplots of physiochemical attributes (pH, Alcoholic Volume, Total Acidity, Volatile Acidity and Total Phenolic Index) and OTA concentration in red wines.

According to OIV [39], five physicochemical parameters from the conventional wine analysis were performed (pH, alcoholic volume, total acidity, volatile acidity, and total phenolic index) on the red wines made from the Xinomavro and Syrah varieties (Figure 1). The pH of Syrah wines was higher (3.52) compared to Xinomavro wines (3.31). The Total Phenolic Index (TPI) showed a similar pattern, with Syrah wines registering greater amounts (48.23) than Xinomavro wines (31.70). There were no appreciable changes in the alcohol content between the Syrah and Xinomavro wines, which were 13.32 and 13.02% (*v*/*v*), respectively. Xinomavro wines had greater amounts of tartaric acid (6.77 mg/L) than Syrah wines (5.72 mg/L) in terms of total acidity. The volatile acidity of Xinomavro and Syrah wines was 0.51 g/L and 0.86 g/L (expressed as acetic acid), respectively, which is consistent with the limits specified in wine regulation.

The OTA concentrations in the red wine samples did not show significant differences between the two red varieties. However, no significant differences were observed in OTA concentration between the red grapevine varieties; Xinomavro presented slightly higher OTA concentration (0.91 μg/L) than the Syrah variety (0.81 μg/L).

**Figure 2:**
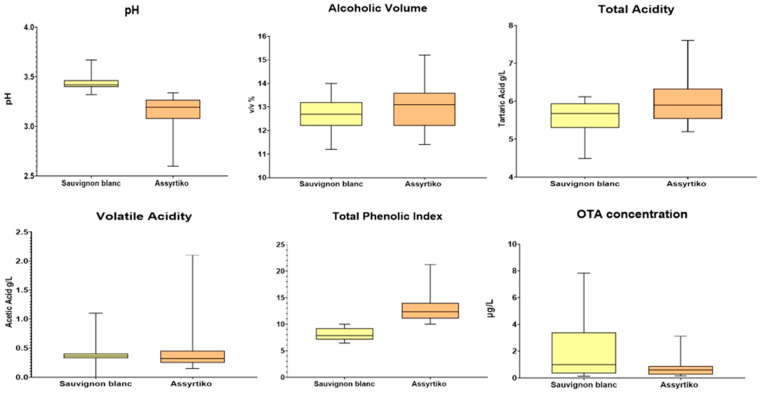
Boxplots of physiochemical attributes (pH, Alcoholic Volume, Total Acidity, Volatile Acidity, and Total Phenolic Index) and OTA concentration in white wines.

Regarding the conventional analyses of the white wines produced by the Assyrtiko and Sauvignon blanc varieties, pH, Alcoholic Volume (% *v*/*v*), Total Acidity (TA, g/L tartaric acid), Volatile Acidity (VA, g/L acetic acid), and Total Phenolic Index (TPI) were performed according to the OIV (2021) (Figure 2). Sauvignon blanc wines showed significantly higher pH value (3.45) compared to Xinomavro wines (3.13).

Moreover, the Alcoholic Volume and TA of the wines produced by the Assyrtiko variety were higher (13.09%, *v*/*v* and 6.12 g/L tartaric acid, respectively) compared to those produced by the Sauvignon blanc variety (12.64%, *v*/*v* and 5.54 g/L tartaric acid, respectively), but no statistical significance was observed between the two varieties (Figure 2). Assyrtiko wines showed the highest TPI levels between the two varieties (Figure 2). As for the volatile acidity, white wines showed no defect since volatile acidity was at a low level.

The OTA concentrations in the analyzed white wines did not show significant differences between the two white varieties. However, Sauvignon blanc presented a higher OTA incidence (2.16 μg/L) than the Assyrtiko variety (0.74 μg/L).

### 3.4. Principal Component Analysis

**Figure 3:**
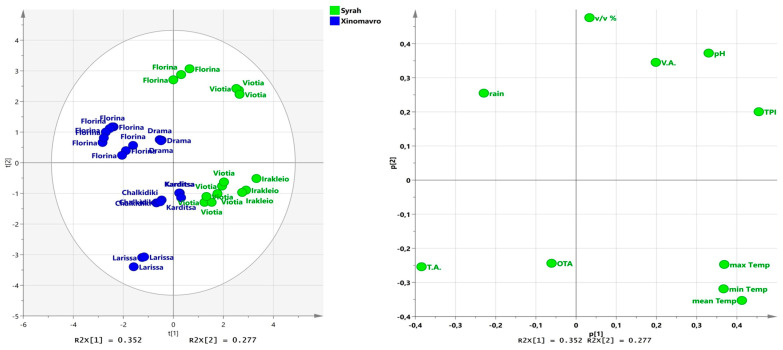
The principal component analysis (PCA) plot of scores (**left**) and correlation loadings (**right**) in the plane of the first two principal components for the red wine varieties Syrah and Xinomavro (T.A.: titratable acidity; V.A.: volatile acidity; TPI: total phenolic index; *v*/*v*%: alcoholic volume; OTA: ochratoxin A concentration; rain: Total rainfall.

**Figure 4:**
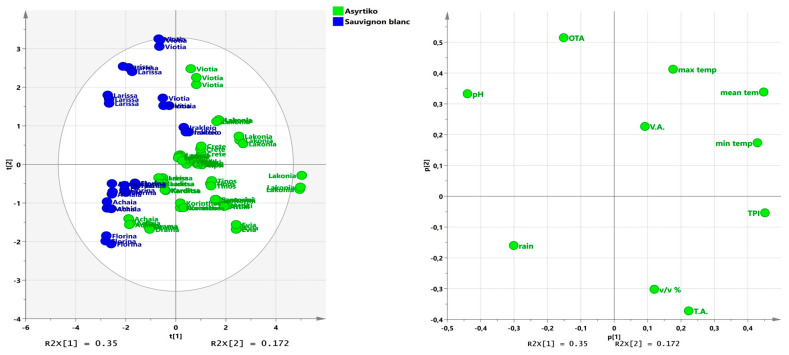
The principal component analysis (PCA) plot of scores (**left**) and correlation loadings (**right**) in the plane of the first two principal components for the white wine varieties Sauvignon blanc and Assyrtiko (T.A.: titratable acidity; V.A.: volatile acidity; TPI: total phenolic index; *v*/*v*%: alcoholic volume; OTA: ochratoxin A concentration; rain: Total rainfall.

The spatial distribution of the 15 weather stations is presented in Supplementary Figure S1. The climatic conditions that were meticulously selected for analysis were specifically those that prevailed during the critical months that lie between the véraison stage of the grapevine variety and the subsequent harvesting period. Fungi, responsible for producing OTA, exhibit their peak activity and mycotoxin synthesis during this particular interval. Two principal components explained approximately 62.9% of the total variability in the red varieties (Figure 3). The first component (PC1) was mostly associated with TPI and rain, explaining approximately 35.2% of the total variance. PC1 was characterized by the contrast between the TPI, which displayed positive loadings, and rainfall levels with negative loadings. The second component (PC2), explaining 27.7% of the remaining variance, was mainly related to OTA concentration, environmental temperature (Tmax, Tmin, Tmean), VA, pH, and TPI. PC2 was characterized by the contrast among alcoholic volume (*v*/*v*, %), pH, VA, and TPI presenting positive loadings, whereas OTA concentration, and Tmax, Tmin, Tmean displayed negative loadings. Regarding the first axis (PC1), wines produced from Florina and Viotia (Syrah variety) vineyards located in northern and central Greece (Supplementary Figure S1) were strongly defined by high levels of alcoholic strength, pH, VA, and TPI, while wines produced at Larissa (Xinomavro variety) were located on the opposite side of PC1 (Figure 4) and were strongly associated with the highest levels of OTA concentrations. Rain was observed to scatter predominantly in the second quadrant, as defined by the positive values of PC2. This suggests a positive correlation with wines produced from Drama and Florina, areas located in northern Greece (Supplementary Figure S1). Climatological conditions such as temperature (Tmin, Tmax and Tmean), were predominantly located in the fourth quadrant with positive values of PC1, indicating a positive correlation between wines produced from Irakleio and Viotia (Supplementary Figure S1).

In the biplot illustrated in Figure 3, two distinct groups were unequivocally identifiable because of the investigation of the conventional parameters analyzed, which included the concentrations of OTA, as well as the various climatological conditions pertinent to the regions where the grapes were cultivated. It must be emphasized that the two distinct groups delineated in the biplot correspond to the two grapevine varieties that were the focus of this study, with the variety Xinomavro positioned on the left side of the biplot and the internationally recognized variety Syrah situated on the right side. Furthermore, wines produced in the regions of Viotia and Irakleio exhibited a positive correlation with increased levels of minimum temperature (Tmin), maximum temperature (Tmax), and mean temperature (Tmean), whereas wines originating from the areas of Florina and Drama demonstrated a significant positive correlation with higher levels of precipitation, as depicted in Figure 4.

For the white grapevine varieties, the two principal components accounted for 52.3% of the total variability (Figure 4). The first component (PC1) was mostly related with Tmin, TPI, and pH, explaining approximately 35% of the total variance (Figure 4). PC1 is characterized by the contrast between Tmin, and TPI, which displays positive loadings and pH with negative loadings. In contrast, the second component (PC2), which explained 17.2% of the remaining variance, was positively related to the OTA concentration and Tmax, while alcoholic volume, rain, and TA presented negative loadings (Figure 4). Regarding the first axis (PC1), wines produced in Laconia, located in the southeastern part of the Peloponnese peninsula, were strongly defined by higher values of TPI, Tmax, Tmin, and Tmean. and with intermediate values of OTA concentrations, whereas wines produced in Achaia and Florina were mostly defined by higher values of rainfall. Regarding the second axis (PC2), two mainland regions, Viotia and Larissa, were characterized by higher OTA levels, pH and Tmax, and were positively associated with PC2, whereas wines produced in Achaia and Florina were characterized by higher rainfall values.

Two groups were distinguished in the biplot of the two PCs because of the similarities in the wine producing areas. On the right side of the plot, the wines produced by the Greek wine variety Assyrtiko are defined by higher levels of TPI, and on the left side are the wines produced by the international variety Sauvignon blanc. Furthermore, wines produced in Achaia and Florina are located in the lower left quarter due to higher rainfall. On the other hand, there is a tendency for the Greek islands (Santorini, Tinos and Crete) and other coastal areas (Laconia) to group together because of their similarly higher Tmax, Tmin, and Tmean (Figure 4).

### 3.5. Correlation Between the Occurrence of OTA and Meteorological Parameters

Many factors influence the presence and concentration of OTA in wine. The climate and geographical origin of grapes or wine have a major impact on OTA contamination [12,35,44,45]. Wine samples and climate data from the grape-growing season (May to September) were collected to investigate the relationship between OTA content and meteorological parameters. Climate data used in our study included monthly mean temperature, monthly maximum temperature, monthly lowest temperature, and rainfall.

Table 3 presents the values of Pearsons’ correlation coefficient (r) and the coefficient of determination (R squared) calculated by the linear regression for each wine variety. Table 3 shows that the OTA concentration was positively correlated with the mean monthly temperature in all varieties. The data obtained revealed a relatively strong correlation of weather conditions with OTA level in analyzed wine samples since all Pearsons’ correlation coefficients were above 0.5. The same trend was followed for maximum and minimum temperature. Regarding the rainfall influence on OTA level, there was almost no linear relationship between the two variables with all Pearson correlation coefficients in the four grapevine varieties, which were below 0.2.

## 4. Discussion

Societal endorsement, emerging from the acknowledgment of the health benefits associated with moderate intake of red wine, could significantly contribute to the establishment of a routine practice of consuming this alcoholic beverage in modest quantities [46]. Conversely, prolonged exposure to even minimal levels of mycotoxins present in wine may result in detrimental health outcomes and potentially culminate in chronic ailments, including oncological conditions [47,48]. Consequently, proficient functionality of the Rapid Alert System for Food and Feed (RASFF) and national health regulatory bodies is of paramount importance to ensure consumer protection. Regulatory entities must promptly identify and mitigate the potential risks associated with xenobiotic contamination of food products. Therefore, robust verification processes for food products are essential. It is imperative to consider the necessity for innovative research endeavors, including the quantification of various mycotoxins in wine and the grapes utilized in wine production, conducted before and during the vinification process at meticulously defined critical control points.

Climate change is a significant contemporary issue, engendering severe repercussions across various domains. A recent analysis conducted by the Intergovernmental Panel on Climate Change (IPCC) [49] indicates that since the pre-industrial era (1850–1900), the average surface temperature has exhibited a continuous increase, attaining approximately 1 °C above pre-industrial benchmarks by 2017 (an average increase of 0.2 °C per decade) as a result of historical and ongoing emissions attributable to anthropogenic activities [49]. The agricultural sector has emerged as one of the sectors most adversely affected by this rise in temperature. Projections suggest that global agricultural output may decline by 6.9% by 2050 as a consequence of climate change [50]. This predicament has been attributed to an increased frequency, intensity, and duration of heat-related phenomena, such as droughts, heatwaves, and alterations in precipitation patterns, which are progressively becoming more common and extreme [51], consequently resulting in both desertification and inundation of agricultural fields. Climate change is recognized as a critical factor influencing the occurrence of ochratoxin A (OTA) in grapes [8] in conjunction with geographical location and phytopathological conditions. Meteorological conditions, harvest time, biotic elements, agricultural practices, and the specific grapevine variety may also exert an influence on the final OTA concentration [3]. With respect to climate change, the ramifications of elevated temperatures have been correlated with increased OTA levels in wines originating from Italy and Spain, with 10% of the samples exhibiting concentrations exceeding 2 μg/L [38]. Earlier research conducted by Soleas et al. [52] identified a correlation between temperature and the prevalence of OTA in both grapes and wines, concluding that wines produced in southern European countries contained higher levels of OTA compared to those from northern Europe.

The systematic observation and evaluation of OTA concentrations within wine products is of paramount significance, primarily because of the notably high prevalence of this mycotoxin in vinous beverages, as well as its well-documented mutagenic properties and toxicological potential, which pose substantial health risks [13,14]. Furthermore, the growing apprehension surrounding climate change necessitates ongoing and rigorous actions aimed at establishing and understanding potential trends related to OTA concentrations in wines over time, considering the various environmental and climatic factors that may influence these levels [38]. Furthermore, the revision of OTA exposure assessment requires continuous monitoring of its prevalence and new risk assessment studies that will consider both the impact of climate change on vineyard ecology and fungal contamination dynamics, as well as wine updated consumption patterns in different populations [18,53]. Empirical evidence indicates that Greek wines originating from regions characterized by warmer climates, as well as those produced in vintages that are climatically unfavorable, exhibit increased concentrations of OTA. It is noteworthy that the frequency of OTA contamination in these wines is not only dependent on geographic factors but is also significantly influenced by the specific grape varieties utilized. A comparative analysis with previous studies conducted in Greece [31,32,33,34,35,36,37] revealed a modest yet discernible increase in OTA levels in wines when assessed on a decadal scale, suggesting a potential trend that warrants further investigation. In the comprehensive analysis of all wines under scrutiny, it was found that the concentrations of OTA present were consistently lower than the Maximum Permissible Limit (MPL) established by the European Union, which is set at 2 μg/L, thereby categorizing these wines as safe for human consumption in accordance with regulatory standards. The contamination of wine by OTA may result from substandard agricultural practices and fungal infestation of the grapes themselves [54]. The prevalence of OTA in vineyard settings is correlated with factors such as temperature, pH, and water activity, along with specific grape varieties [8,55]. Fungi that produce OTA can grow on substrates with a moisture content ranging from 10% to 20%, with an optimal growth temperature of approximately 25–30 °C [56].

In this study, the occurrence of OTA was examined in single varietal wines originating from the Greek region, specifically from the red cultivars Syrah and Xinomavro, and the white varieties Sauvignon blanc and Assyrtiko.

A comprehensive sample of 72 commercial wines was analyzed, of which the presence of OTA was detected in 40 samples (55%). Among these, 7 samples (9.7%) exhibited concentrations that exceeded the legally permissible limit (2 μg/L). Notably, 3 of these samples were marginally above this threshold. Surprisingly in our research, OTA was observed more frequently in white wines than in red wines compared to other research studies [6,11,57] according to the differentiation of the winemaking process [58]. Within the Sauvignon blanc variety, OTA was particularly prevalent (83%), with this variety demonstrating the highest concentrations, reaching 7.59 μg/L and yielding an average value of 2.16 μg/L. The average OTA concentrations of the other analyzed varieties were significantly lower, with Xinomavro presenting the highest (0.94 μg/L), followed by Syrah (0.8 μg/L) and Assyrtiko (0.73 μg/L). Only one sampled wine of the Xinomavro variety found slightly above the EU established Maximum Permissible Limit (MPL) for OTA (2.09 μg/L). The Sauvignon blanc variety is characterized by a relatively thin skin compared to both red and other white varietals [59]. Consequently, if the fungal pathogen colonizes the skin, thinness facilitates penetration into the interior of the grape. Our experimental findings exhibited significant discrepancies when compared to those reported in other investigations, potentially attributable to the substantial temporal gap between the studies, utilization of divergent measurement techniques, and variations in the origins and types of wine analyzed. Numerous studies have also monitored fluctuations in OΤA concentration throughout the vinification process. According to these findings, OTA levels tend to decrease during the fermentation phase [10,60,61]. Moreover, there was a long interval between the research mentioned above and the present study. During this period, climate changes and agriculture practices may have affected the structure of black aspergilli population and resulted in the better adaption of *A. tubingensis* and *A. uvarum* species in the agroecosystem of Greece.

Between the two white grape varieties that were rigorously examined in this study, namely Assyrtiko and Sauvignon blanc, a statistically significant difference in the concentration of OTA was observed, which may be attributed to the comparatively higher phenolic content found in Assyrtiko in relation to other white varietals within the viticultural landscape [61,62]. This observation can be further supported by the fact that the berries of the Assyrtiko variety have a notably thicker skin in comparison to other Greek white grape varieties with the resultant wines exhibiting higher TPI, which renders the colonization of fungi more challenging [63,64].

Lower frequencies of OTA incidence were recorded in the red than in white grape varieties. This observation appeared to be associated with the interaction between anthocyanins and OTA. Specifically, during the initial phase of red vinification, where skins, seeds, stems, and must are combined, an ionic bond is formed between anthocyanins and OTA, leading to their complexation and subsequent precipitation [65]. This interaction is facilitated by the pH of the wine, which ranges from 3 to 3.5, in conjunction with the pKa of the carboxyl group of the phenylalanine moiety of OTA being 4.4, whereas that of anthocyanins is approximately 3 [66]. Consequently, the toxin undergoes partial dissociation, resulting in a positive charge on the amine group (NH_3_^+^), whereas anthocyanins transfer the negative charge to the oxygen of the hydroxyl groups [61,64]. With regard to the wine samples analyzed, it was observed that wines with a high phenolic index did not either contain detectable levels of OTA or exhibited very low concentrations. Conversely, wines characterized by low phenolic content demonstrated a higher frequency of OTA. Additionally, it is pertinent to mention that thin-skinned varietals are typically not abundant in phenolic constituents [67].

In the context of conventional wine analyses, there appears to be a notable absence of any statistically significant correlation when examining the relationships among alcoholic strength, pH levels, and total acidity in relation to the presence of ochratoxin A (OTA), a mycotoxin of considerable concern in oenological studies. The volatile acidity determined in the wines was at low levels, suggesting that these wines do not manifest any defects that would compromise their quality. Despite the lack of significant correlations, some discernible trends were readily observable during the analysis. For example, it was noted that the Assyrtiko and Syrah wines exhibited markedly higher TPI levels than the Sauvignon blanc and Xinomavro wines, whereas the pH levels measured for Syrah and Sauvignon were comparatively higher when juxtaposed with those of Xinomavro and Assyrtiko, although the differences were not statistically significant. The findings from the chemical analyses of the samples revealed minor fluctuations among the wines, a phenomenon attributable to the winemaking protocols employed by each winery, as well as the intrinsic characteristics of each grape variety.

It is widely acknowledged that wine is significantly influenced by the quality of the grapes, which is contingent upon climatic conditions. While variations in the biochemical conditions of grapes are critical, it is noteworthy that identical wines can be consistently produced year after year through adjustments in industrial winemaking techniques. Conversely, the issue of mycotoxin contamination presents considerable challenges. Recent research has elucidated the climatic variables, particularly temperature and humidity, that are pivotal for the proliferation of toxigenic fungi and the subsequent production of ochratoxin A (OTA) by these organisms during pre-harvest, post-harvest, and transportation phases. The optimal ecological parameters for fungal growth have been established at temperatures ranging from 30 to 37 °C [45,68], whereas the production of OTA occurs optimally within a temperature range of 15 to 25 °C [69,70]. Detrimental growth conditions, especially fluctuations in temperature, may induce fungi to synthesize a variety of secondary metabolites, including OTA. In conjunction with the findings of Bellí et al. [7], our research suggests that temperatures exceeding the optimal growth range substantially promote fungal proliferation and aggravate OTA production. Detrimental growth conditions, especially fluctuations in temperature, may induce fungi to synthesize a variety of secondary metabolites, including OTA. Another factor affecting the presence of OTA seems to be the location, as a more frequent occurrence of the toxin was observed in regions with a warmer climate, such as central Greece (Viotia), than in cooler regions, such as Florina. Specifically, the highest concentrations of OTA were found in Thebes for Assyrtiko, Sauvignon, and Syrah, and in Larissa for Xinomavro. High average annual temperatures occurred during the years concerned with wine production, favoring the growth of the fungus *Aspergillus carbonarius*. In the areas where the lowest OTA concentrations were noted, the average annual temperature did not vary significantly, but the annual rainfall was significantly lower.

Climate change is not just about warming, but also about changing precipitation patterns, and extreme weather events, all of which profoundly affect the fungus–grape interaction. More especially concerning warmer growing seasons, *A. carbonarius* is a thermotolerant fungus. Rising average temperatures, particularly during the veraison-to-harvest period, create more favorable conditions for its growth and OTA production [71]. The optimal temperature for OTA production by *A. carbonarius* is around 25–30 °C [4]. Regions previously considered too cool for significant OTA risk (e.g., parts of Northern Europe, higher altitude areas) are now becoming more susceptible. Conversely, traditionally high-risk, hot regions (e.g., parts of the Mediterranean) may become so hot and dry that fungal growth is inhibited, potentially shifting the highest risk to intermediate, warming regions. Regarding changes in precipitation patterns and drought stress, grapes present higher sugar concentration and thinner skins, making them more susceptible to fungal invasion and insect damage [72]. Furthermore, intense rain or hail events close to harvest can cause berry damage and create massive spiking in OTA risk by providing multiple infection sites. Climate change can alter the prevalence of insect pests (e.g., the Vine Moth, *Lobesia botrana*) whose larvae could damage grape berries [73]. This damage is a primary entry point for *Aspergillus* fungi. Warmer winters may lead to higher insect populations, further increasing the risk. For wine producers in high-risk regions, the changing climate introduces significant practical implications, including increased frequency of OTA contamination and substantial economic repercussions. Contaminated batches may face rejection or require costly remediation, directly impacting profitability. Furthermore, there is a risk of reputational damage to a brand, as consumers and importers demand consistently safe products. This evolving threat means that producers can no longer rely solely on historical risk data and must instead invest in new management and monitoring strategies to protect their wine and their business.

The susceptibility of grapes to ochratoxin A (OTA) contamination is influenced not only by external factors, such as climatic conditions, but also by intrinsic varietal characteristics. Grape variety and its genetically determined morphological traits, particularly a compact cluster architecture (berry arrangement), are critical risk factors. Such compact shapes create a microclimate with restricted air flow and higher humidity, conditions that promote the growth of OTA-producing fungi, predominantly *Aspergillus* species. Furthermore, the degree of ripeness at harvest is critical, as overripe berries with weakened skin integrity and higher sugar content provide a more readily available substrate for fungal colonization and toxin production. This interaction between genetic predisposition, reflected in cluster structure, and the physiological condition of the berry has been established as a key determinant of OTA accumulation in the resulting wine [42,45,64].

To effectively reduce OTA contamination, a holistic approach is essential. Pre-harvest mitigation begins in the vineyard with careful canopy management to improve air circulation and the judicious use of irrigation to prevent berry splitting [74]. The most critical step is selective harvesting, which involves training crews in order to avoid damaged grapes and, where possible, employing optical sorting technology [75] at the winery reception to mechanically remove diseased and desiccated berries—a primary source of the toxin. Post-harvest, minimizing skin contact time and using specific fining agents like activated carbon can further reduce the final OTA levels, though the latter must be applied cautiously to avoid stripping wine quality. A robust, forward-looking monitoring and surveillance program is the key to climate resilience [12]. This necessitates a shift from merely testing at harvest to implementing a predictive, risk-based system. Producers should begin surveillance during véraison, correlating local weather data with OTA risk models and using in-field rapid tests to screen high-risk blocks. Analytically, investing in sensitive quantification methods is crucial for accurate low-level detection. Ultimately, industry-wide collaboration to share anonymized data on OTA levels and weather patterns will be key to refining predictive models and safeguarding regional reputations in the face of a volatile climate.

Another reason for the intensification of controls, followed by free availability of prevalence data, are the recent findings of scarce but valuable studies on the cumulative risk from multiple mycotoxin presence in a food commodity. The added toxicity from the presence of more than one mycotoxin raises this demand on multitoxin presence data, while will help towards a more complete risk assessment for the effects of OTA to human health due to wine consumption [76].

In addition to the contribution of the present work to the assessment of the levels of OTA contamination of wines produced in the Greek territory, the development of an analytical method is important. Specifically, a modified protocol of the QuEChERS method was used, as in Testempasis et al. [37], for the extraction of OTA from wines. This method is quick, simple and economical, and allows for the determination and quantification of OTA in wines, as it shows good sensitivity and appropriate precision, as established by the recovery of the standard solutions for a range of OTA concentrations. The results obtained during the validation process demonstrated sufficient accuracy and repeatability. This shows the potential of applying quantitative determination of OTA in wines, as it is a much more economical solution.

## 5. Conclusions

A more comprehensive and overarching conclusion that can be synthesized from the extensive analysis presented in this study is that the rate of contamination by ochratoxin A (OTA) within wines originating from the Greek geographical territory is observed to be considerably elevated, thereby necessitating a robust framework for ongoing monitoring and evaluation in order to safeguard public health and ensure the integrity of the oeno- viticultural sector. The research findings delineated in this study exhibit a significant degree of comparability with prior investigations that have been conducted within the same geographical context of Greece, as evidenced by the scholarly contributions of Markaki et al. [31], Stefanaki et al. [32], Soufleros et al. [33], Salaha et al. [34], Labrinea et al. [35], Sarigiannis et al., [36] and Testempasis et al. [37], all of which provide a foundational basis for understanding the recurring nature of this issue. It is imperative to recognize that the environmental conditions which govern agricultural production are increasingly unpredictable, a phenomenon that can be directly attributed to the far-reaching impacts of climate change, which in turn has profound implications for the prevalence of mycotoxins within various food products. The elevated temperatures that have become a hallmark of contemporary climatic patterns, particularly during the summer months, appear to create a favorable environment that not only facilitates the proliferation but also promotes the colonization of fungi that are capable of producing OTA.

It is particularly noteworthy that our findings indicate that substantial quantities of OTA can be synthesized under a wide range of climatic conditions. Due to the capacity of OTA to be produced across a broad spectrum of temperatures, it is feasible for OTA to be continuously generated in the field, especially during the harvest period, despite the presence of considerable day-to-night temperature fluctuations. Additional climatic factors, such as precipitation, may also exert a significant influence on the contamination of wine with OTA, as increased rainfall promotes the proliferation of OTA-producing black Aspergilli.

## Figures and Tables

**Table 1:** Analytical method performance.

Analyte	Matrix	LOD (μg/L)	LOQ (μg/L)	Recovery Range (%)	RSDr (%) *
Ochratoxin A	Wine	0.03	0.01	85–94	2.5–3.5

* RSDr (%) = Relative standard deviation.

**Table 2:** Incidence of Ochratoxin A in the wines (concentrations in μg/L).

Variety	Total Samples	Positive Samples	Positive Samples (%)	Positive Samples > 2 μg/L	Min	Max	Median	Average	STDV
Syrah	12	5	41	0	0.171	1.857	0.591	0.804	0.656
Xinomavro	23	7	30	1	0.288	2.078	0.652	0.930	0.591
Sauvignon blanc	12	10	83	5	0.149	7.587	1.188	2.160	2.427
Assyrtiko	25	18	72	2	0.160	2.523	0.601	0.744	0.639

**Table 3:** Correlation between meteorological parameters and Ochratoxin A concentration of wines using the coefficients of Pearson.

Variety	Correlation	OTA Concentration vs. Mean Temp	OTA Concentration vs. Max Temp	OTA Concentration vs. Min Temp	OTA Concentration vs. Rain
Syrah	R	0.663	0.776	0.499	0.251
	R squared	0.440	0.603	0.249	0.063
	*p* value	0.070	0.0007	0.058	0.366
Xinomavro	R	0.860	0.648	0.832	−0.432
	R squared	0.739	0.430	0.6937	0.187
	*p* value	0.0001	0.001	0.0001	0.050
Sauvignon blanc	R	0.639	0.449	0.7080	0.043
	R squared	0.409	0.201	0.501	0.001
	*p* value	0.0001	0.012	0.0001	0.821
Assyrtiko	R	0.488	−0.050	0.501	0.126
	R squared	0.234	0.002	0.251	0.016
	*p* value	0.0002	0.718	0.0001	0.361

## Data Availability

The data presented in this study are available on request from the corresponding authors (pending privacy and ethical considerations).

## Supplementary Materials

The following supporting information can be downloaded at: https://www.mdpi.com/article/10.3390/foods14234157/s1, Figure S1: Map of Greece showing the 15 wine producing locations used in the current research; Table S1: Mean ochratoxin A (OTA), concentrations (μg/L) in different varieties of red wine and climatological condition of the wine producing areas (concentrations above EU Maximum Permissible Limit—MPL are highlighted with bold font); Table S2: Mean ochratoxin A (OTA), concentrations (μg/L) in different varieties of white wine and climatological condition of the wine producing areas (concentrations above EU Maximum Permissible Limit—MPL are highlighted with bold font).

## References

[1] FAO-OIV (2019). 2019 Statistical Report on World Vitiviniculture.

[2] Freire L., Passamani F.R.F., Thomas A.B., Nassur R.D.C.M.R., Silva L.M., Paschoal F.N., Pereira G.E., Prado G., Batista L.R. (2017). Influence of physical and chemical characteristics of wine grapes on the incidence of Penicillium and Aspergillus fungi in grapes and ochratoxin A in wines. Int. J. Food Microbiol..

[3] Gil-Serna J., Vázquez C., González-Jaén M.T., Patiño B. (2018). Wine contamination with ochratoxins: A review. Beverages.

[4] Jørgensen K. (2005). Occurrence of ochratoxin A in commodities and processed food–A review of EU occurrence data. Food Addit. Contam..

[5] European Commission (2002). Reports on Tasks for Scientific Cooperation. Report of Experts Participating in Task 3.2.7. Assessment of Dietary Intake of Ochratoxin A by the Population of EU Member States. Directorate-General Health and Consumer Protection. https://food.ec.europa.eu/system/files/2016-10/cs_contaminants_catalogue_ochratoxin_task_3-2-7_en.pdf.

[6] Zimmerli B., Dick R. (1996). Ochratoxin A in table wine and grape-juice: Occurrence and risk assessment. Food Addit. Contam..

[7] Bellí N., Bau M., Marín S., Abarca M.L., Ramos A.J., Bragulat M.R. (2006). Mycobiota and ochratoxin A producing fungi from Spanish wine grapes. Int. J. Food Microbiol..

[8] Paterson R.R.M., Venâncio A., Lima N., Guilloux-Bénatier M., Rousseaux S. (2018). Predominant mycotoxins, mycotoxigenic fungi and climate change related to wine. Food Res. Int..

[9] Anli E., Bayram M. (2009). Ochratoxin A in wines. Food Rev. Int..

[10] Lasram S., Mani A., Zaied C., Chebil S., Abid S., Bacha H., Mliki A., Ghorbel A. (2008). Evolution of ochratoxin A content during red and rose vinification. J. Sci. Food Agric..

[11] Battilani P., Magan N., Logrieco A. (2006). European research on ochratoxin A in grapes and wine. Int. J. Food Microbiol..

[12] Quintela S., Villarán M.C., López de Armentia I., Elejalde E. (2013). Ochratoxin A removal in wine: A review. Food Control.

[13] El Khoury A., Atoui A. (2010). Ochratoxin A: General Overview and Actual Molecular Status. Toxins.

[14] IARC, International Agency for Research on Cancer (1993). Some Naturally Occurring Substances: Food Items and Constituents, Heterocyclic Aromatic Amines and Mycotoxins. IARC Monographs on the Evaluation of Carcinogenic Risks to Humans.

[15] EFSA (European Food Safety Authority) (2006). Opinion of the Scientific Panel on Contaminants in the Food Chain (CONTAM) on a request from the Commission related to Ochratoxin A in Food, Question N° EFSA-Q-2005-154, Adopted on 4 April 2006. EFSA J..

[16] IARC, International Agency for Research on Cancer (2012). Risk Assessment and Risk Management of Mycotoxins. IARC Sci. Publ..

[17] EFSA, European Food Safety Authority (2025). The Four Steps of Risk Assessment. https://multimedia.efsa.europa.eu/riskassessment/index.htm.

[18] Annunziata L., Campana G., De Massis M.R., Aloia R., Scortichini G., Visciano P. (2025). Ochratoxin A in Foods and Beverages: Dietary Exposure and Risk Assessment. Expo. Health.

[19] European Commission (2023). Commission Regulation (EU) 2023/915 of 25 April 2023 on maximum levels for certain contaminants in food and repealing Regulation (EC) No 1881/2006 (Text with EEA relevance). Off. J. Eur. Union.

[20] Karsauliya K., Yahavi C., Pandey A., Bhateria M., Sonker A.K., Pandey H., Sharma M., Singh S.P. (2022). Co-occurrence of mycotoxins: A review on bioanalytical methods for simultaneous analysis in human biological samples, mixture toxicity and risk assessment strategies. Toxicon.

[21] Rizzazzi-Fazeli E., Reiter E.V., De Saeger S. (2011). Sample preparation and clean up strategies in the mycotoxin analysis: Principles, applications and recent developments. Determining Mycotoxins and Mycotoxigenic Fungi in Food and Feed.

[22] Fabiani A., Corzani C., Arfelli G. (2010). Correlation between different clean-up methods and analytical techniques performances to detect ochratoxin A in wine. Talanta.

[23] Huertas-Perez J.F., Arroyo-Manzanares N., Garcia-Campaña A.M., Gamiz-Gracia L. (2016). Solid-phase extraction as sample treatment for the determination of ochratoxin A in foods: A review. Crit. Rev. Food Sci. Nutr..

[24] Al-Taher F., Banaszewski K., Jackson L., Zweigenbaum J., Ryu D., Cappozzo J. (2013). Rapid method for the determination of multiple mycotoxins in wines and beers by LC-MS/MS using a stable isotope dilution assay. J. Agric. Food Chem..

[25] Arroyo-Manzares N., Garcia-Campaña A.M., Gamiz-Gracia L. (2011). Comparison of different sample treatments for the analysis of ochratoxin A in wine by capillary HPLC with laser-induced fluorescence detection. Anal. Bioanal. Chem..

[26] Mariño-Repizo L., Gargantini R., Manzano H., Raba J., Cerutti S. (2016). Assessment of ochratoxin A occurrence in Argentine red wines using a novel sensitive QuEChERS-solid phase extraction approach prior to ultra-high performance liquid chromatography-tandem mass spectrometry methodology. J. Sci. Food Agric..

[27] Pizzutti I.R., de Kok A., Scholten J., Righi L.W., Cardoso C.D., Rohers G.N., da Silva R.C. (2014). Development, optimization and validation of a multimethod for the determination of 36 mycotoxins in wines by liquid chromatography-tandem mass spectrometry. Talanta.

[28] Li H., Wang H., Li H., Goodman S., van der Lee P., Xu Z., Fortunato A., Yang P. (2018). The worlds of wine: Old, new and ancient. Wine Econ. Policy.

[29] Merkouropoulos G., Miliordos D.E., Tsimbidis G., Hatzopoulos P., Kotseridis Y. (2023). How to Improve a Successful Product? The Case of “Asproudi” of the Monemvasia Winery Vineyard. Sustainability.

[30] Miliordos D.E., Merkouropoulos G., Kogkou C., Arseniou S., Alatzas A., Proxenia N., Hatzopoulos P., Kotseridis Y. (2021). Explore the Rare—Molecular Identification and Wine Evaluation of Two Autochthonous Greek Varieties: “Karnachalades” and “Bogialamades”. Plants.

[31] Markaki P., Delpont-Binet C., Grosso F., Dragacci S. (2001). Determination of Ochratoxin A in Red Wine and Vinegar by Immunoaffinity High-Pressure Liquid Chromatography. J. Food Prot..

[32] Stefanaki I., Foufa E., Tsatsou-Dritsa A., Dais P. (2003). Ochratoxin A concentrations in Greek domestic wines and dried vine fruits. Food Addit. Contam..

[33] Soufleros E.H., Tricard C., Bouloumpasi E.C. (2003). Occurrence of ochratoxin A in Greek wines. J. Sci. Food Agric..

[34] Salaha M.J., Metafa M., Lanaridis P. (2007). Ochratoxin A occurrence in Greek dry and sweet wines. OENO One.

[35] Labrinea E.P., Natskoulis P.I., Spiropoulos A.E., Magan N., Tassou C.C. (2011). A survey of ochratoxin A occurrence in Greek wines. Food Addit. Contam. Part B.

[36] Sarigiannis Y., Kapolos J., Koliadima A., Tsegenidis T., Karaiskakis G. (2014). Ochratoxin A levels in Greek retail wines. Food Control.

[37] Testempasis S.I., Kamou N.N., Papadakis E.N., Menkissoglu-Spiroudi U., Karaoglanidis G.S. (2022). Conventional vs. organic vineyards: Black Aspergilli population structure, mycotoxigenic capacity and mycotoxin contamination assessment in wines, using a new Q-TOF MS-MS detection method. Food Control.

[38] De Jesus C.L., Bartley A., Welch A.Z., Berry J.P. (2018). High Incidence and Levels of Ochratoxin A in Wines Sourced from the United States. Toxins.

[39] International Organization of Vine and Wine (OIV) (2025). Compendium of International Methods of Analysis of Wines and Musts.

[40] Lagouvardos K., Kotroni V., Bezes A., Koletsis I., Kopania T., Lykoudis S., Mazarakis N., Papagiannaki K., Vougioukas S. (2017). The automatic weather stations network of the National Observatory of Athens (NOANN): Operation and database. Geosci. Data J..

[41] Hocking A.D., Leong S.L., Kazi B.A., Emmett R.W., Scott E.S. (2007). Fungi and mycotoxins in vineyards and grape products. Int. J. Food Microbiol..

[42] Leong S.L., Hocking A.D., Pitt J.I., Kazi B.A., Emmett R.W., Scott E.S. (2006). Australian research on ochratoxigenic fungi and ochratoxin A. Int. J. Food Microbiol..

[43] Gambuti A., Strollo D., Genovese A., Ugliano M., Ritieni A., Moio L. (2005). Influence of enological practices on ochratoxin A concentration in wine. Am. J. Enol. Vitic..

[44] Pietri A., Bertuzzi T., Pallaroni L., Piva G. (2001). Occurrence of ochratoxin A in Italian wines. Food Addit. Contam..

[45] Belli N., Marın S., Sanchis V., Ramos A.J. (2004). Influence of wateractivity and temperature on growth of isolates of Aspergillus section Nigri obtained from grapes. Int. J. Food Mictobiol..

[46] Weiskirchen S., Weiskirchen R. (2016). Resveratrol: How much wine do you have to drink to stay healthy?. Adv. Nutr..

[47] Chunmei J., Junling S., Qian H., Yanlin L. (2013). Occurrence of toxin-producing fungi in intact and rotten table and wine grapes and related influencing factors. Food Control.

[48] Marin S., Ramos A.J., Cano-Sancho G., Sanchis V. (2013). Mycotoxins: Occurrence, toxicology, and exposure assessment. Food Chem. Toxicol..

[49] IPCC, Intergovernmental Panel on Climate Change (2018). Global Warming of 1.5 °C. An IPCC Special Report on the Impacts of Global Warming of 1.5 °C Above Pre-Industrial Levels and Related Global Greenhouse Gas Emission Pathways, in the Context of Strengthening the Global Response to the Threat of Climate Change, Sustainable Development, and Efforts to Eradicate Poverty. https://www.ipcc.ch/site/assets/uploads/sites/2/2019/06/SR15_Full_Report_High_Res.pdf.

[50] Wiebe K., Lotze-Campen H., Sands R., Tabeau A., van der Mensbrugghe D., Biewald A., Bodirsky B., Islam S., Kavallari A., Mason-D’Croz D. (2015). Climate change impacts on agriculture in 2050 under a range of plausible socioeconomic and emissions scenarios. Environ. Res. Lett..

[51] Meehl G.A., Stocker T.F., Collins W.D., Friedlingstein P., Gaye T., Gregory J.M., Zhao Z.C., Solomon D.S., Qin M., Manning Z., Chen M., Marquis K.B., Averyt M., Tignor H.L., Miller (2007). Global climate projections. Climate Change 2007: The Physical Science Basis.

[52] Soleas G.J., Yan J., Goldberg D.M. (2001). Assay of ochratoxin A in wine and beer by high pressure liquid chromatography photodiode array and gas chromatography mass selective detection. J. Agric. Food Chem..

[53] Schrenk D., Bodin L., Chipman J.K., del Mazo J., Grasl-Kraupp B., Hogstrand C., Hoogenboom L., Leblanc J.C., Nebbia C.S., Nielsen E. (2020). Scientific Opinion on the risk assessment of ochratoxin A in food. EFSA J..

[54] Liew W.P.P., Mohd-Redzwan S. (2018). Mycotoxin: Its impact on gut health and microbiota. Front. Cell. Infect. Microbiol..

[55] Zhang H., Apaliya M.T., Mahunu G.K., Chen L., Li W. (2016). Control of ochratoxin A-producing fungi in grape berry by microbial antagonists: A review. Trends Food Sci. Technol..

[56] Klarić M.Š., Rašić D., Peraica M. (2013). Deleterious effects of mycotoxin combinations involving ochratoxin A. Toxins.

[57] Otteneder H., Majerus P. (2000). Occurrence of ochratoxin A (OTA) in wines: Influence of the type of wine and its geographical origin. Food Addit. Contam..

[58] Battilani P., Silva A., Reynolds A.G. (2010). Controlling ochratoxin A in the vineyard and winery. Managing Wine Quality.

[59] Robinson J., Harding J., Vouillamoz J. (2012). Wine Grape. A Complete Guide to 1368 Vine Varieties, Including Their Origins and Flavours.

[60] Anli R.E., Vural N., Bayram M. (2011). Removal of ochratoxin A (OTA) from naturally contaminated wines during the vinification process. J. Inst. Brew..

[61] Dachery B., Hernandes K.C., Veras F.F., Schmidt L., Augusti P.R., Manfroi V., Zini C.A., Welke J.E. (2019). Effect of Aspergillus carbonarius on ochratoxin A levels, volatile profile and antioxidant activity of the grapes and respective wines. Food Res. Int..

[62] Bayman P., Baker J.L. (2006). Ochratoxins: A global perspective. Mycopathologia.

[63] Veras F.F., Dachery B., Manfroi V., Welke J.E. (2020). Colonization of Aspergillus carbonarius and accumulation of ochratoxin A in Vitis vinifera, Vitis labrusca, and hybrid grapes–research on the most promising alternatives for organic viticulture. J. Sci. Food Agric..

[64] Jiang C., Shi J., Zhu C. (2013). Fruit spoilage and ochratoxin a production by Aspergillus carbonarius in the berries of different grape cultivars. Food Control.

[65] Freire L., Braga P.A., Furtado M.M., Delafiori J., Dias-Audibert F.L., Pereira G.E., Reyes F.G., Catharino R.R., Sant’Ana A.S. (2020). From grape to wine: Fate of ochratoxin A during red, rose, and white winemaking process and the presence of ochratoxin derivatives in the final products. Food Control.

[66] Lee H.J., Kim H.D., Ryu D. (2024). Practical strategies to reduce ochratoxin A in foods. Toxins.

[67] Flamini R., Mattivi F., De Rosso M., Arapitsas P., Bavaresco L. (2013). Advanced knowledge of three important classes of grape phenolics: Anthocyanins, stilbenes and flavonols. Int. J. Mol. Sci..

[68] Leong S.L., Hocking A.D., Pitt J.I. (2004). Occurrence of fruit rot fungi (Aspergillus section Nigri) on some drying varieties of irrigated grapes. Aust. J. Grape Wine Res..

[69] Esteban A., Abarea M.L., Bragnlat M.R., Cabanes F.J. (2004). Effects of temperature and incubation time on production of ochratoxin A by blace Aspergilli. Res. Microbiol..

[70] Mitchell D., Parra R., Aldred D., Magan N. (2004). Water and temperature relation of growth and ochratoxin A production by Aspergillus carbonarius strains from grapes in Europe and Israel. J. Appl. Microbiol..

[71] Clouvel P., Bonvarlet L., Martinez A., Lagouarde P., Dieng I., Martin P. (2008). Wine contamination by ochratoxin A in relation to vine environment. Int. J. Food Microbiol..

[72] Kassemeyer H.-H. (2017). Fungi of grapes. Biology of Microorganisms on Grapes, in Must and in Wine.

[73] Ioriatti C., Anfora G., Bagnoli B., Benelli G., Lucchi A. (2023). A review of history and geographical distribution of grapevine moths in Italian vineyards in light of climate change: Looking backward to face the future. Crop Prot..

[74] Cameron W., Petrie P.R., Bonada M. (2024). Effects of vineyard management practices on winegrape yield components. A review using meta-analysis. Am. J. Enol. Vitic..

[75] Hendrickson D.A., Lerno L.A., Hjelmeland A.K., Ebeler S.E., Heymann H., Hopfer H., Block K.L., Brenneman C.A., Oberholster A. (2016). Impact of mechanical harvesting and optical berry sorting on grape and wine composition. Am. J. Enol. Vitic..

[76] Abdullah R., Kamarozaman N.S., Ab Dullah S.S., Aziz M.Y., Aziza B.A. (2025). Health risks evaluation of mycotoxins in plant-based supplements marketed in Malaysia. Sci. Rep..

