# Supplementary material for: Impact of Climate Change on the Presence of Ochratoxin A in Red and White Greek Commercial Wines
Source: Foods. 2025 Dec 3;14(23):4157. doi: 10.3390/foods14234157 (PMC12691814; doi:10.3390/foods14234157)
Supplement: Supplementary file 1 [file foods-14-04157-s001.zip › foods-3932958-supplementary.pdf]

foods MDPI

Supplementary Materials

Impact of Climate Change on the Presence of Ochratoxin A in Red and White Greek Commercial Wines

Dimitrios Evangelos Miliordos [1,2], Lamprini Roussi [2,3], Stamatina Kallithraka [2], Efstathios Z. Panagou [3] and Pantelis I. Natskoulis [1,*]

1 Institute of Technology of Agricultural Products, Hellenic Agricultural Organisation (ELGO)—DIMITRA, 1 Sofokli Venizelou, 14123 Likovrisi, Greece
2 Laboratory of Oenology and Alcoholic Drinks, School of Food and Nutritional Sciences, Department of Food Science & Human Nutrition, Agricultural University of Athens, 75 Iera Odos, 11855 Athens, Greece
3 Laboratory of Microbiology and Biotechnology of Foods, School of Food and Nutritional Sciences, Department of Food Science and Human Nutrition, Agricultural University of Athens, 75 Iera Odos, 11855 Athens, Greece

* Correspondence: p.natskoulis@elgo.gr

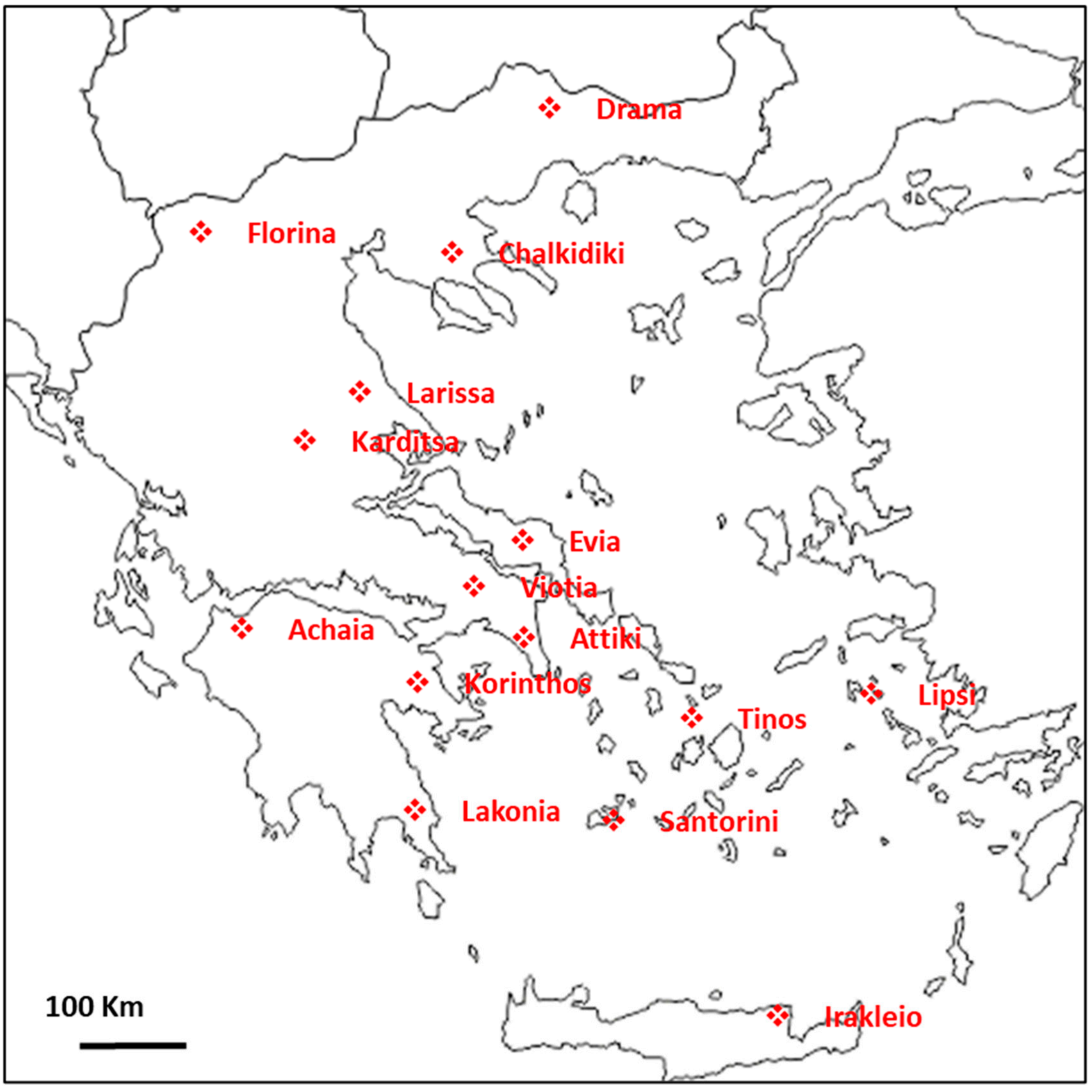

Supplementary Figure S1. Map of Greece showing the 15 wine producing locations used in the current research.

Supplementary Table S1. Mean ochratoxin A (OTA), concentrations (μg/L) in different varieties of red wine and climatological condition of the wine producing areas (concentrations above EU Maximum Permissible Limit—MPL are highlighted with bold font)

Variety	Area	Vintage	OTA (μg/L)	Mean Temp. (°C)	Maximum Temp. (°C)	Minimum Temp. (°C)	Rainfall (mm)
Xinomavro	Chalkidiki	2021	1.395±0.049	23.5	33.92	15.36	17.78
Xinomavro	Larissa	2020	**2.078±0.100**	23.9	37.06	13.4	24.56
Xinomavro	Florina	2020	0.546±0.015	19.8	34	8.5	52.6
Xinomavro	Florina	2020	0.630±0.049	22.3	35.58	10.48	52.6
Xinomavro	Drama	2021	0.563±0.028	19.6	34.5	8.6	32.14
Xinomavro	Karditsa	2021	0.288±0.027	19.76	34.2	8.56	24.88
Xinomavro	Florina	2021	0.930±0.066	22.3	36.58	10.48	16.2
Syrah	Viotia	2022	0.241±0.028	20.6	35.04	14.4	23.44
Syrah	Florina	2021	0.591±0.079	23.8	36.58	7.54	16.2
Syrah	Viotia	2023	1.160±0.079	24.16	38.34	13.76	49.44
Syrah	Viotia	2020	1.857±0.089	25.04	38.5	16.4	13.44
Syrah	Irakleio	2021	0.171±0.007	23.88	37.12	12.22	4.68

Supplementary Table S2. Mean ochratoxin A (OTA), concentrations (μg/L) in different varieties of white wine and climatological condition of the wine producing areas (concentrations above EU Maximum Permissible Limit—MPL are highlighted with bold font)

Variety	Area	Vintage	OTA (μg/L)	Mean Temp (°C)	Maximum Temp. (°C)	Minimum Temp. (°C)	Rainfall (mm)
Sauvignon blanc	Florina	2022	1.188±0.056	22.1	34.77	12.65	60.3
Sauvignon blanc	Larissa	2023	**3.271±0.287**	22.9	35.02	12.54	69.66
Sauvignon blanc	Florina	2020	**2.255±0.450**	20.58	34.78	9.86	55.92
Sauvignon blanc	Larissa	2022	**5.247±0.269**	24.16	38.34	13.76	66.38
Sauvignon blanc	Viotia	2022	**2.899±0.252**	25.04	37.18	16.4	23.44
Sauvignon blanc	Viotia	2022	**7.587±0.060**	24.16	38.34	13.76	23.44
Sauvignon blanc	Achaia	2023	0.667±0.031	20.44	34.34	7.78	29.68
Sauvignon blanc	Achaia	2022	0.749±0.081	20.64	32.78	10.74	72.4
Sauvignon blanc	Irakleio	2023	0.150±0.005	22.96	36.92	11.42	4.68
Sauvignon blanc	Florina	2023	0.149±0.006	19.86	32.76	9.34	66.55
Assyrtiko	Larissa	2021	0.626±0.054	23.90	38.54	11.66	34.96
Assyrtiko	Larissa	2022	0.733±0.143	22.96	36.92	11.42	66.38
Assyrtiko	Attiki	2020	0.589±0.030	24.57	33.12	18.87	15.33
Assyrtiko	Korinthos	2020	0.571±0.010	22.46	37.12	9	30.8
Assyrtiko	Santorini	2020	0.915±0.052	23.82	33.24	18.5	0.44
Assyrtiko	Karditsa	2020	1.016±0.065	25.3	38.12	19.6	64.36
Assyrtiko	Drama	2020	0.937±0.058	25.3	35.58	14.7	118.76
Assyrtiko	Larissa	2021	0.323±0.058	24.25	38.22	14.67	34.98
Assyrtiko	Evia	2022	0.181±0.115	22.05	35. 85	13.65	15.7
Assyrtiko	Lipsi	2020	0.633±0.035	24.55	32.125	19.22	8.12
Assyrtiko	Lakonia	2021	0.207±0.007	23.35	36.125	16.60	38.25

Assyrtiko	Lakonia	2021	0.184±0.009	23.35	36.125	16.60	38.25
Assyrtiko	Crete	2022	**2.143±0.111**	24.42	33.325	17.32	39.12
Assyrtiko	Lakonia	2021	0.753±0.031	23.35	36.125	16.60	38.25
Assyrtiko	Viotia	2022	**2.523±0.566**	26.35	38.125	19.675	32.02
Assyrtiko	Attiki	2023	0.313±0.032	24.05	35.725	15.7	22.27
Assyrtiko	Tinos	2023	0.196±0.032	23.76	35.73	14.63	19.95
Assyrtiko	Achaia	2022	0.583±0.093	20.64	32.78	10.74	72.4
